# Estimating medical risk in human spaceflight

**DOI:** 10.1038/s41526-022-00193-9

**Published:** 2022-03-31

**Authors:** Erik L. Antonsen, Jerry G. Myers, Lynn Boley, John Arellano, Eric Kerstman, Binaifer Kadwa, Daniel M. Buckland, Mary Van Baalen

**Affiliations:** 1 Center for Space Medicine, Department of Emergency Medicine, Baylor College of Medicine, Houston, TX 77007 USA; 2 NASA Glenn Research Center, Cleveland, OH 44135 USA; 3 KBR, Houston, TX 77058 USA; 4 Aegis Aerospace Inc., Houston, TX 77058 USA; 5 Department of Preventive Medicine and Population Health, University of Texas Medical Branch, Galveston, TX 77555 USA; 6 NASA Johnson Space Center, Houston, TX 77058 USA; 7 Duke University, Durham, NC 27708 USA

**Keywords:** Occupational health, Risk factors, Epidemiology

## Abstract

NASA and commercial spaceflight companies will soon be retuning humans to the Moon and then eventually sending them on to Mars. These distant planetary destinations will pose new risks—in particular for the health of the astronaut crews. The bulk of the evidence characterizing human health and performance in spaceflight has come from missions in Low Earth Orbit. As missions last longer and travel farther from Earth, medical risk is expected to contribute an increasing proportion of total mission risk. To date, there have been no reliable estimates of how much. The Integrated Medical Model (IMM) is a Probabilistic Risk Assessment (PRA) Monte-Carlo simulation tool developed by NASA for medical risk assessment. This paper uses the IMM to provide an evidence-based, quantified medical risk estimate comparison across different spaceflight mission durations. We discuss model limitations and unimplemented capabilities providing insight into the complexity of medical risk estimation for human spaceflight. The results enable prioritization of medical needs in the context of other mission risks. These findings provide a reasonable bounding estimate for medical risk in missions to the Moon and Mars and hold value for risk managers and mission planners in performing cost-benefit trades for mission capability and research investments.

## Introduction

With the exception of the U.S. Apollo missions, the history of human spaceflight has exclusively occurred in low Earth orbit (LEO). The longest lunar mission performed to date was Apollo 17; three astronauts spent 12 days traveling to and from the moon; two astronauts spent 75 h on the lunar surface^1^. Because of astronauts’ experience on long-duration spaceflight in LEO, the deconditioning effects of missions out to 6 months are generally well characterized. Significant unknowns remain, however, regarding the resilience of the human system during sustained lunar and Mars mission durations.

With the bulk of prior operational experience in LEO, it is easy for mission planners to make faulty assumptions regarding the magnitude of human system risk. In LEO, the risk from human system failure, or medical risk, has historically been small compared to the risk involved in getting to and from space^2^. As mission duration extends for sustained lunar and Mars missions, this risk balance shifts. Medical risk in a mission is strongly correlated to both distance from Earth and mission duration. This paper is designed to improve understanding and communication of medical risk in human spaceflight. Characterizing medical risk is part of addressing the question of whether astronauts will become ill or injured during a given mission and, by extension, whether they will be able to perform the jobs they are asked to do in those missions. This paper is the first publication of this type of medical probabilistic risk assessment (PRA) comparing evidence-based quantitative estimates of spaceflight medical risk across the spectrum of mission durations that NASA uses for planning purposes. As both government and commercial spaceflight consider LEO, lunar, and Mars missions over the next decade, an understanding of the changing risk to human health and performance associated with increased distance from Earth and mission duration is critical to mission success.

LEO operations include specific advantages in the medical risk arena that contribute to a low medical risk posture when compared to missions beyond LEO. As mission duration increases, deconditioning effects on the human body become more pronounced. In addition, distance from Earth creates three key operational changes that increase medical risk^3^:

1. Communication: Medical support in LEO depends on real-time communications to enable flight surgeons to provide telemedical evaluation and recommendations quickly and accurately. When real-time communications are no longer possible, the operational paradigm shifts to store-and-forward telemedicine. In this paradigm, the crew must act autonomously during the initial phases of any medical issue^4,5^.
2. Resupply: Medical support also depends on a robust consumables resupply chain that exists in LEO. This resupply chain will be strained for lunar missions and non-existent for consumables on a Mars mission. This does not imply that pre-positioning of needed resources will not occur, but degradable resources like food and medications are not amenable to that model and this can alter the medical risk posture^6–9^.
3. Evacuation: The option for evacuation of an ill or injured crewmember to definitive care is feasible in LEO in a reasonable timeframe. In lunar missions, evacuation times are longer which implies that a different set of medical capabilities may be required to stabilize an ill or injured crewmember for a much longer transfer time. In the case of a Mars mission, evacuation to definitive care is unavailable. These differences suggest that the current operating paradigm for medical support will need to change to mitigate increased risk^3^. The effective design and implementation of a Crew Health and Performance (CHP) System that anticipates these changes is important to mission success.

NASA has used the Integrated Medical Model (IMM) to inform International Space Station and other probabilistic risk assessment (PRA) based mission analyses^10^. However, estimates of medical risks for exploration spaceflight and comparison with past risk acceptance are lacking in the literature. This is in part because of unique challenges associated with modeling medical outcomes in human spaceflight. With fewer than 600 people having flown in space under a wide range of conditions and non-standardized monitoring and medical tracking across those missions, the evidence base needed to inform medical risk analysis is still evolving. NASA uses Design Reference Missions (DRMs) that provide a baseline set of mission assumptions to enable common risk assessment information^11^. Existing medical PRA approaches that were developed for LEO have limitations when applied to future missions beyond LEO. However, these types of PRA applications can still provide value in helping mission planners approach a reasonable order of magnitude and bounding for risk. IMM is used here to estimate medical risk differences between DRMs that represent past, current, and future NASA and commercial missions under consideration. The results are then considered in the context of evidence surrounding the effects of the operational changes from LEO discussed above and the effects of long-duration spaceflight on the deconditioning of humans.

## Methods

The IMM was developed at NASA starting in 2008 and was transitioned to operations in 2017^12^. It is used here to provide quantitative risk assessment for each of the DRMs considered below. IMM models 100 medical conditions and includes the capability to assess the impact of resource limitation or depletion on successful treatment of medical conditions. Full description of the model and its validation is provided elsewhere in the literature^10,12–14^.

Table 1 shows the DRMs considered for analysis including mission duration and number of crew. These are chosen based on the average durations and crew complements for existing missions or current planning for future missions. These durations and crew complements may change in future mission design iterations and so should be considered an approximation here. The model simulates two DRMs that reflect previous mission types including Space Shuttle (DRM 1) and the International Space Station (DRM 4). The remaining DRMs approximate potential future missions for which NASA needs risk assessment. DRMs 2 and 3 approximate short-duration LEO or lunar missions. DRM 5 approximates longer-duration LEO or lunar missions and Mars preparatory missions. DRMs 6 and 7 approximate initial and sustaining Mars missions.

**Table 1:** Design reference mission attributes.

DRM	Mission duration (days)	Number of crew
1	14	7
2	21	4
3	42	4
4	180	6
5	365	4
6	730	4
7	1195	4

The IMM incorporates evidence from all ISS missions as well as data from Apollo, Skylab, Mir, and Space Shuttle programs, but the resource table is baselined to the ISS medical system resources^10^. Medical capability here is defined as the complete set of resources that enable the crew to perform medical monitoring, diagnosis, and treatment for medical conditions that occur in spaceflight. Resources needed for treatment are explicit in the IMM resource table, but resources such as monitoring capabilities, diagnostic capabilities, and crew capability implicitly reflect the ISS operating paradigm in the outcomes data that feed the model. The ISS medical capability is comprehensive and rigorously scrutinized; it is specifically intended to provide options for minimizing LEO spaceflight medical risk^10^. The ISS medical capability is assumed here to represent a reasonable upper bound on the benefits that medical resources are likely to bring to the risk posture. This is because of increasing mass, volume, power, and data bandwidth restrictions that are expected to limit medical resources in missions that occur beyond LEO. This assumption could be challenged by advances in autonomous medical capability or propulsion technology that may address mass and volume limits, for example, future enhanced capabilities not currently available may result in more risk reduction for less mass and volume.

For the purpose of comparing the effectiveness of the fielded medical capability for each of the DRMs, we model in units of ‘ISS Medical Capability’^10^ described below:

1. No Medical Capability—this case approximates a mission scenario where there is completely ineffective matching of medical capability to medical need within the mission or no resources available. In this case all conditions go untreated.
2. Unlimited ISS Medical Capability—this case approximates the best possible matching between medical capability and medical need that could be expected, based on the historic ISS Medical Capability. In Unlimited ISS Medical Capability, all conditions are modeled as fully treated.
3. Limited ISS Medical Capability—this case represents a tailorable example case of no resupply, i.e., an ISS medical capability that can run out of medications. Medical conditions for which there are insufficient resources to fully treat are modeled as partially treated or untreated.

This paper evaluates several mission level outcomes: Total medical events (TME); loss of crew life (LOCL), likelihood of reaching consideration of crewmember evacuation criteria (EVAC), and crew health index (CHI). (Note: The one hundred medical conditions modeled by IMM are shown in Extended Data in Table 2). The incidence of IMM medical events occurring during simulated missions is based on historical mission and cohort data contained within the Integrated Medical Evidence Database (iMED)^14,15^. For each condition, the probable percentage of occurrence of “best case” and “worst case” scenarios are specified, as well as a defined set of medical resources that are used to treat the condition. Best case conditions are those that present in the mild-moderate end of the clinical spectrum. Worst cases are those that present on the severe end of the clinical spectrum^10^. The iMED entry for each condition details the specific medical resources and quantity necessary for treatment in both the best and worst-case scenarios. In-flight medical treatment is assumed to follow a specified protocol or clinical practice guidelines for each medical condition and is constrained by resource availability on the ISS (i.e., the ISS Medical Capabilities)^10^.

**Fig. 1:**
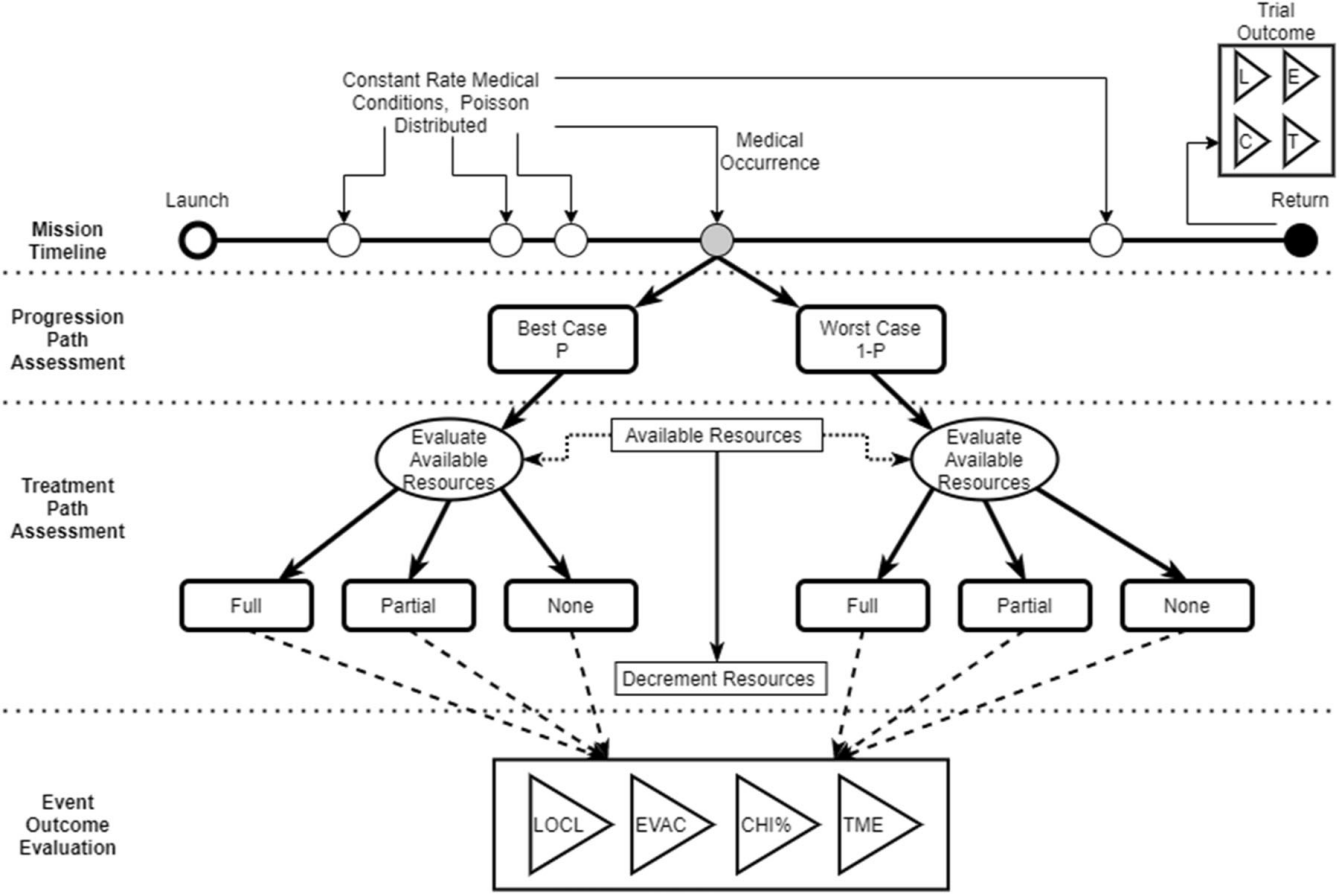
The IMM 4.1 Monte-Carlo simulation incorporates a mission timeline, progression-path assessment, treatment-path assessment, and event-outcome evaluation. These are summed across all occurring conditions to provide a trial outcome and across all simulation runs to provide the simulation outcomes.

The IMM modeling process is shown in Fig. 1 below. It is described in detail elsewhere and is briefly summarized here^10,12–14^.

IMM uses stochastic processes via Monte-Carlo simulation. First mission and crew characteristics are specified for a given DRM. Each IMM Monte-Carlo iteration includes medical condition occurrence along the Mission Timeframe that progress to a Best or Worst Case scenario in the Progression Path Assessment Step. Best and worst-case scenarios for medical conditions are generated based on probability distributions. Incidence of medical conditions is held constant. During the Treatment Path Assessment step, available resources are queried and either Full, Partial, or No treatment is applied to the specific condition. Medical events, treatments, and outcomes are randomly generated based on probability distributions. Event Outcomes are evaluated and tallied to provide a trial outcome. Summation of the health and medical outcomes across all simulation iteration steps provides the primary outcomes^10^. Primary outcomes for this study include TME, CHI, EVAC, LOCL, and required resources.

CHI is a calculated percentage using the quality-adjusted mission time lost (QAMTL) due to in-flight medical events and resources available to treat those conditions.

1 $${{{\mathrm{CHI}}}} = \left( {1 - \left( {{{{\mathrm{QAMTL}}}} \div {{{\mathrm{Mission}}}}\;{{{\mathrm{Length}}}}} \right)} \right) \times {{{\mathrm{100}}}}$$

For a given condition, QAMTL is determined by summing the product of functional impairment and duration for three clinical phases of that condition: (1) diagnosis and initial treatment, (2) ongoing treatment, and (3) end-state. Functional impairment (FI) is an estimated measure of the affected crewmember’s health and performance ability. An average for the entire crew and the mission are reported. CHI percent values range from 0–100, where zero represents complete crew impairment and 100 represents a completely functional crew. Functional impairments in the IMM are estimated using the AMA Guides to the Evaluation of Permanent Impairment’s general principles and rules^16^. Note that the AMA Guides estimate permanent impairment based on terrestrial norms. Application of these assessments in estimating functional impairment in a spaceflight environment likely overpredict impairment in some outcomes (i.e., lower limbs) and underpredict impairment in other instances (i.e., eyes, hands) Given that comprehensive space environment functional impairment data has not been collected, the AMA Guides are considered a sufficiently robust evidence source for making relative medical risk assessments.

EVAC in the context of IMM means that medical evacuation from the ISS is considered for definitive treatment of the afflicted crewmember. When this outcome is reached, it effectively ends the mission for that crewmember. Here ‘definitive’ treatment is defined as the best possible treatment available at a US tertiary care hospital on Earth. EVAC is considered an end-state result if any of the following criteria are met: (1) potential LOCL; (2) potential significant permanent impairment; or (3) potential intractable pain. When an EVAC state is reached during an IMM simulation, the availability of a return vehicle or the likelihood of a successful clinical outcome should a return vehicle be available is assumed to exist.

LOCL in the context of IMM is interpreted to mean that the clinical scenario resulted in death of the affected crewmember(s). During the simulation, the rarity of the LOCL and EVAC end-states results in crewmembers only assigned one end-state of EVAC or LOCL in most simulation trials. In rare instances, EVAC or LOCL for a second condition is reached prior to being reached for an earlier condition. Both events are recorded as EVAC or LOCL outcomes.

Required resources for treatment are tracked for each simulated mission trial. The initial quantities for medical resources are baselined to a fully stocked ISS medical kit. The quantity of each resource used to treat a given condition is decremented from available resources. If more of any particular resource is used than is available, then that resource is considered “depleted” in the Limited ISS Medical Capability scenario. Events that require a “depleted” resource are considered “partially treated” if only a portion of the required resources are available. If there are sufficient available alternate resources in the Single ISS Medical Capability scenario, the event is considered “fully treated.” If there are no resources available to treat the event, the event is considered “untreated.” When an event is “partially treated”, outcomes are based on a weighted average of the treated and untreated values. For example, if a condition has a 0% chance of going to EVAC in the best case treated scenario and a 0–100% chance of going to EVAC in the best case untreated scenario, then under best case partial treatment, it is possible the condition will go to EVAC.

Each IMM simulation consists of 100,000 Monte-Carlo trials, where each trial is considered a unique mission simulation. Convergence of each simulation is evaluated by confirming a <5% change in the average standard deviation of the CHI, EVAC, and LOCL model outcomes in the last 2 sets of 1000 simulation mission trials.

CHI is calculated as a percentage, with 95% confidence intervals (CIs) for the associated distributions. EVAC and LOCL are probabilities, with 95% CI of the mean for EVAC and LOCL obtained using bootstrap resampling of the simulation output.

Definition of crew attributes allow for tailoring crew-dependent variations by defining a limited set of individual factors that affect medical illness. These include sex, coronary artery calcium score, dental crowns, contact lens use, and prior abdominal surgery. The model does not consider most crew attributes that are already attenuated by the astronaut selection standards and flight certification standards^17^. Environmental Injury likelihood is attributed in part to individual crewmembers engaging in extravehicular activity, where decompression sickness, paresthesias, and fingernail delamination are medical conditions linked to EVA. Note that Tables 3–5 documenting the assigned individual attributes by crewmember used for each DRM are shown in Extended Data.

### Reporting summary

Further information on research design is available in the  Nature Research Reporting Summary linked to this article.

## Results

**Fig. 2:**
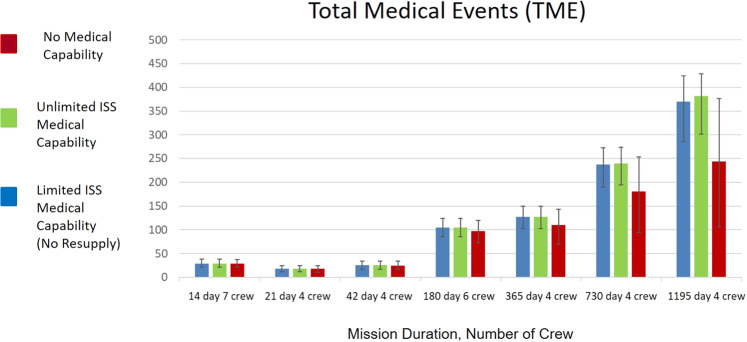
Total medical events predicted for the indicated mission duration and crew complement. The results are shown by DRM across the x-axis and subdivided by the level of medical capability included in each situation. The 95% CIs is interpreted as the uncertainty estimated with respect to the data informing the 100 medical conditions and the PRA abstraction of the medical event timeline and end-states. As unaccounted uncertainties are not represented in this formulation or data representation, outcomes should be considered as representative of relative changes in risk rather than absolute risk assessments^14^. ISS International Space Station.

Results below are organized to show the IMM outputs for each DRM listed in Table 1 and subdivided for each DRM by the cases of medical capability modeled. Figure 2 illustrates the estimated TME that are likely to be encountered by crew with 95% CI. The number of predicted medical events range from <20 in mission durations <180 days, to many hundreds for mission durations exceeding 180 days, regardless of the crew compliment size used in each simulation.

The number of TME is useful to help medical capabilities designers understand an approximate scope of need for a medical system. Medical systems that must deal with a small number of conditions are likely to be designed differently than those requiring more capability. The graph above shows that missions with no medical systems have the same or a lower number of crew medical events as missions with ISS medical capabilities. This is not because the crew are healthier. On the contrary, it is because crew die or are evacuated over the course of the mission, lowering the total number of crew that complete the mission. Thus, at the end of an 1195 day mission, in many cases the model is measuring likely medical events not for a crew of 4, but for a crew of 2 or 3.

**Fig. 3:**
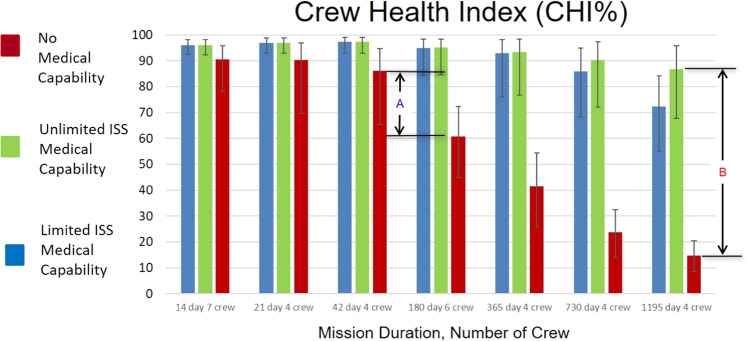
Crew health index (CHI) predicted for the indicated mission duration and crew complement. 100 represents fully functional crew while 0 represents totally incapacitated crew. Parameters A and B are discussed in the text. The results are shown by DRM across the x-axis and subdivided by the level of medical capability included in each situation. The 95% CIs is interpreted as the uncertainty estimated with respect to the data informing the 100 medical conditions and the PRA abstraction of the medical event timeline and end-states. As unaccounted uncertainties are not represented in this formulation or data representation, outcomes should be considered as representative of relative changes in risk rather than absolute risk assessments^14^. ISS International Space Station.

The effect of medical conditions on crew readiness and task performance is approximated by the CHI parameter as shown in Fig. 3. Here the y-axis ranges from zero to 100%, where 100% represents fully functional crew and zero indicates totally incapacitated crew.

The ‘No Medical Capability’ case exhibits a substantial decrease in CHI as missions exceed 42 days shown by the marker ‘A’ in Fig. 3. For DRMs 6 and 7, the ‘Unlimited ISS Medical Capability’ and ‘Limited ISS Medical Capability’ perform similarly. Crew size in DRMs <180 days shows no dependence on resupply as the ‘Unlimited ISS Medical Capability’ and ‘Limited ISS Medical Capability’ cases do not diverge.

The marker ‘B’ shown in Fig. 3 highlights the difference for a given mission and crew profile between the ‘Unlimited ISS Medical Capability’ and the ‘No Medical Capability’ cases. This suggests that for an 1195 day mission with a crew of 4, CHI could range from ~15% up to ~88% depending on the effectiveness of the medical capability designed into the mission. For shorter-duration missions the difference is smaller. This is discussed further in the discussion section below.

**Fig. 4:**
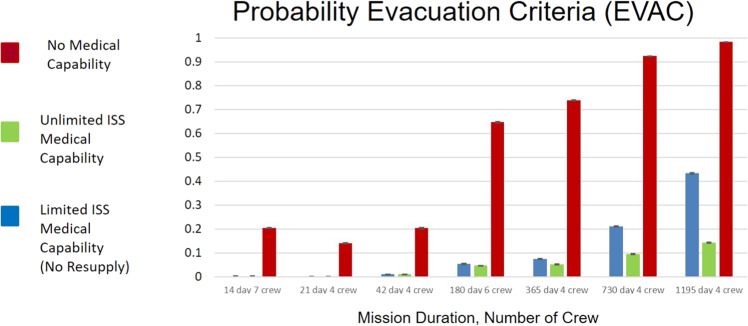
Calculated likelihood that crew would consider evacuation if it were available due to medical conditions experienced in-flight. The 95% CIs is interpreted as the uncertainty estimated with respect to the data informing the 100 medical conditions and the PRA abstraction of the medical event timeline and end-states. As unaccounted uncertainties are not represented in this formulation or data representation, outcomes should be considered as representative of relative changes in risk rather than absolute risk assessments^14^. ISS International Space Station.

Figure 4 shows the predicted DRM differences in the probability that LEO evacuation criteria will be met. The results suggest that the missions simulated with duration <42 days will have between 0.3 and 1% likelihood of EVAC even with Unlimited ISS Medical Capability provided. In the case of ‘No Medical Capability’, EVAC is a significant concern, exceeding 10% likelihood. These results show >90% likelihood of EVAC in missions exceeding 730 days for ‘No Medical Capability and >10% even in the case of ‘Unlimited ISS Medical Capability’. Although the ‘No Medical Capability’ case is unlikely in actual operations, it can help quantitatively bound and provide insight into the potential range of risk mitigation that medical capability design can bring to a DRM. This is helpful in defining the medical trade space when considering vehicle and system design priorities.

**Fig. 5:**
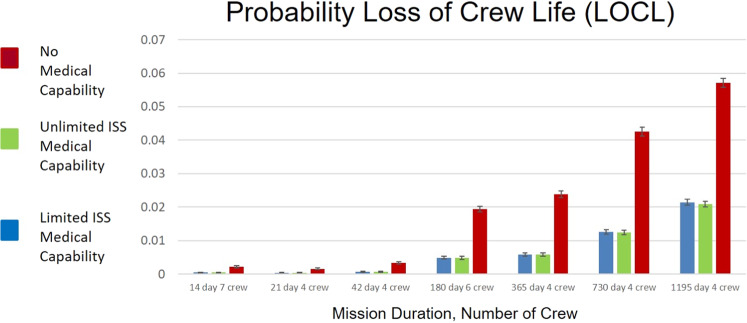
Calculated likelihood that loss of crew life (LOCL) will occur in-mission due to medical conditions experienced in-flight. The 95% CIs is interpreted as the uncertainty estimated with respect to the data informing the 100 medical conditions and the PRA abstraction of the medical event timeline and end-states. As unaccounted uncertainties are not represented in this formulation or data representation, outcomes should be considered as representative of relative changes in risk rather than absolute risk assessments^14^. ISS International Space Station.

Figure 5 shows the difference in probability of LOCL for the same set of missions considered above. It is important to note that these results show LOCL likelihood is around an order of magnitude smaller than the EVAC likelihood. These results show trends similar to the EVAC results for ‘No Medical Capability’, where likelihood of LOCL is more than double that of ‘Limited ISS Medical Capability’ or ‘Unlimited ISS Medical Capability’. Unlike EVAC results, the LOCL results show almost no difference between ‘Unlimited ISS Medical Capability’ and ‘Limited ISS Medical Capability’. This suggests that the dependence of LOCL on resupply is small for the DRMs and medical capabilities considered. LOCL is significantly reduced with even ‘Limited ISS Medical Capability’. For 730 and 1195 day DRMs, risk is unlikely to be reduced below 0.01 likelihood even with ‘Unlimited ISS Medical Capabilities’. Note that Tables 6–9 in Extended Data show tabular data for Figs. 2–5, respectively.

## Discussion

The utility of these results is in their potential to help frame and bound mission planning considerations. As systems engineers consider relative prioritization in trade space analyses, the scope of medical capabilities should be commensurate with the amount of risk that must be mitigated to enable a successful mission. Because of this, it is critical to communicate the limitations, assumptions, and unimplemented capabilities, of this approach. This discussion section bridges the divide between appropriate model interpretation and the operational/clinical application of these results for mission planning purposes.

Figure 3 shows two key items that mission planners should consider. The parameter marked ‘A’ in the diagram shows the drop in CHI% between the 42-day and 180-day missions predicted by the model for the ‘No Medical Capability’ case. The 42-day case uses only 4-crew and the 180-day case uses 6 crew, as a result, they are not directly comparable. The 4-crew case at 365 and 730 days follow the expected relative change. This suggests that, within the limits of this study, for shorter-duration missions, the stringent medical criteria for astronaut selection provide meaningful risk mitigation. As missions extend beyond several months, the results suggest that the relative benefits from selecting very healthy people do not mitigate the potential for more and varied medical events with increasing time. The model suggests that the relative contribution to significant risk reduction tips away from that provided by selection benefit somewhere between 42 and 180 days into a mission.

However, medical risk mitigation is also influenced by the design and inclusion of effective medical capability. The parameter marked ‘B’ in Fig. 3 shows the difference in CHI between the ‘Unlimited ISS Medical Capability’ case and the ‘No Medical Capability’ case for the 1195 day, 4-crew DRM. This same difference is observed in Figs. 4 and 5 for EVAC and LOCL, respectively. The large difference observed between these extreme cases implies that the level of risk the mission will experience depends on the effectiveness of the medical capability that is included in the CHP system. Effectiveness means that the medical capability provided matches with actual mission medical needs. Beyond 42 days, to improve medical risk posture to an acceptable level, mission planners must prioritize the implementation of an appropriately designed medical capability to best mitigate reductions in crew performance during the mission.

Figure 5 shows the probability of LOCL for each of the DRMs. It is worth noting for comparison that Loss of Crew (LOC) risk that crews faced and the Agency accepted at the end of the Space Shuttle Program was 1/90^18^. Simulated missions with 4 crew beyond 730 days indicate a likelihood of LOCL higher than that level even in the case ‘Unlimited ISS Medical Capability’. This provides context to the expected contribution of medical risk to an eventual Mars mission. Mission planners should consider the risks vs. benefits of investing in medical capabilities that address those situations that lead to LOCL.

### Model limitations

The limitations of the model results as presented are considered here. The term ‘Limitations’ means specifically model limitations that apply when the model is used as designed. In this case, the interpretation of all these results is most appropriate when applied to the mission durations and crews described for LEO DRMs. When considered in that context, the following limitations are important.

First, the evaluation of CHI is premised on one-to-one scaling of the quality time lost (QTL) from analogous permanent impairment injuries tables. These assumptions are expected to result in generally conservative estimates of QTL and may underpredict or overpredict decrements to CHI as the permanent impairment tables are Earth gravity-based and hence in microgravity an injury to the lower extremity may be less impairing than when under 1-g loading. These analyses are also limited in that a partial set of demographics and crew number are analyzed. Full evaluation of the effect these factors have on the evaluation of the mission duration outcomes is unknown. It is expected that the mean assessments would not substantially deviate from those presented here, although the variance of the estimates would likely be larger.

As mentioned in the scientific literature, IMM treats each medical condition effectively as occurring independently from other conditions. In this case, the simulation does not include progression of one condition to another, like urinary tract infection (UTI) to sepsis. This means the total medical events may be over-estimated by this analysis. For example, if progression of a UTI to sepsis is considered a single medical event by mission planners. In addition, for these simulations IMM assumes that every diagnosis and treatment are 100% effective.

While the model includes medical resources that have been present on the ISS, those resources primarily focus on medical treatment capability. There are some basic monitoring capabilities included such as blood pressure cuffs and thermometers, but there are no failure modes for such instruments and the effectiveness of a full CHP system is not considered by the model.

Validation assessment of the IMM model utilized four years of ISS data and data from 20 shuttle missions, held back from the model training data to assess the predictive capability of the PRA system. The results indicated that the model somewhat over predicts medical event occurrence in longer-duration missions of 180 days or more^19^ and underpredicts shorter-duration missions^14^. This suggests that the model may overpredict medical risk as mission duration increases. Another limitation of the model is the need to update the evidence based on data from spaceflight or recently published medical advances that may modify the outcomes. These are expected to materially alter the risk estimates over time.

### Interpretation of results

IMM was designed to characterize risk and inform operations in LEO. Because of this and the fact that almost all the data that informs medical condition incidences is based on LEO missions, the results shown here best approximate risk for missions performed in LEO. Despite these limitations, mission planners will seek to understand what these results mean for risk estimation of upcoming lunar and Mars missions based on duration and crew size. Indeed the 4-crew complements are chosen to represent expected crew sizes for upcoming Lunar and Mars missions. Any attempted extrapolation of these results to future missions operating beyond LEO requires thoughtful consideration.

To apply IMM results to missions beyond LEO, it is important to step outside the model-specific limitations and assumptions and consider unimplemented capabilities when interpreting model results. Here unimplemented capability does not mean that the model has a capability and we simply turned it off. Rather it describes risk assessment capabilities that were never designed into the model but whose omission are seen as a limitation by the clinical, operational, and mission planning communities when considering model results.

For example, IMM does not model long-term health conditions that occur post mission or post career from spaceflight exposure. This is an important consideration for both government and commercial spaceflight providers. The results from IMM reflect in-mission risk only and do not address any medical conditions resulting from launch or landing of the mission vehicle. Decrements in performance are approximated by CHI. However, CHI does not extend to a calculable loss of mission objectives or possible loss of mission for any of these DRMs. Therefore, CHI is a proxy for overall performance decrements due to medical conditions alone. For the purpose of results shown here, it is reasonable to surmise that as the CHI approaches zero, the likelihood of Loss of Mission Objectives increases because crew will be unable to perform their mission tasks.

To interpret in-mission medical risk in the context of other unimplemented capabilities, we return to several of the claims in the introduction. Namely that key operational differences between LEO and beyond LEO missions will affect medical risk as distance from Earth increases, and that deconditioning effects on the human body will affect medical risk as mission duration increases.

The first operational limitation noted in the introduction was loss of real-time communications. The IMM outcomes assume that all medical conditions are perfectly diagnosed and perfectly treated; this is not a reflection of real-world operations. In the case of LEO medical operations for both the Space Shuttle and ISS, diagnosis and treatment of medical issues in-mission are assisted by flight surgeons in real-time and at weekly virtual private medical conferences. Although physician-astronauts are not always present in-flight, they have been present intermittently and likely have affected the data used to inform the model to an unknown extent. Specialists from mission control guide astronauts in real-time through medical and research procedures for both diagnosis and treatment. Though the loss of real-time communications cannot be modeled by IMM, it is clear that the loss of this real-time support will at best have a neutral effect on outcomes and more likely have a negative effect on medical risk posture for Mars DRMs by decreasing the likelihood of timely and appropriate diagnosis decisions and treatment implementation.

The second operational limitation is loss of resupply. The use of units of ‘ISS Medical Capability’ is a convenience metric based on the extensive experience base with ISS and the alignment of the resource database with ISS medical resources. In this case, it allows some assessment of the effect that resupply limitations could have on model outcomes of interest. For most short DRMs, the difference in risk estimated for a Limited vs Unlimited ISS Medical Capability is negligible. Divergence between these medical capability levels in the 730 day and 1195 day duration missions ranges from 5 to 20% for CHI and 100 to 180% for EVAC. This suggests that loss of resupply in a long-duration mission will materially affect medical risk through CHI decrements and EVAC. The relationship with LOCL is less clear when those mission durations are interpreted as a Mars mission.

The case of ‘Unlimited ISS Medical Capability’ represents the most comprehensive medical capability that has flown to date in human spaceflight with effectively no resupply issues. Mass, power, volume, and data bandwidth available for missions beyond LEO become more limited as distance from Earth increases^3,5,11^. This means that there is less room available for medical hardware and consumables. Medical hardware includes diagnostic tools such as ultrasound and treatment tools such as defibrillators while consumables include things like pharmaceuticals and bandages. Decreasing mass, power, and volume results in a decreased envelope for all of these medical system components, meaning that tools that assist with accurate diagnosis may not be included while at the same time diagnostic support from mission control is decreasing due to loss of real-time communications. Loss of resupply limits or removes the ability to replace consumables that have been used or degraded. There is evidence that some pharmaceuticals degrade faster in the spaceflight environment than they do on Earth^6,7,20^. Pharmaceuticals and food are both examples of consumable challenges that may not be solved by pre-positioning of supplies^6,8^. These both affect medical risk posture. Because of these challenges, loss of resupply is likely to worsen medical risk posture.

The third operational limitation was the loss of evacuation capability. Figure 4 shows the results for the mean likelihood of meeting evacuation criteria. In the case of an extended mission durations, the 730 and 1195 day mission scenarios (DRM 6 and 7) approximate Mars mission durations. The extrapolation of this risk estimate to a Mars mission risk estimate is challenging because no evacuation is considered feasible in an actual Mars mission. This limitation of model extrapolation is important to consider in the interpretation of model outcomes. The simulation removes modeled crewmembers from generating further medical events. They do not go to LOCL and they do not use further resources. In a real-world situation, unlike the modeled cases, reaching similar criteria does not result in immediate crew evacuation. Instead, the crew and mission control weigh aspects of mission and crew safety to ascertain the possibility and necessity of a safe evacuation. A safe evacuation would also likely remove additional crewmembers with the ill or injured crewmember. However, the model here removes only the evacuated crewmember from contributing further medical events to the simulation. If all the modeled mission durations were explored as if the mission only takes place in LEO, then evacuation would be considered possible on the same timeframe as ISS experiences and follow similar likelihoods determined in this study. If these same mission durations are planned in cis-lunar space as preparation for a Mars mission, then evacuation is expected to be possible, but a different discussion of the risks and benefits of evacuation must occur due to increased evacuation time back to Earth. The potential requirement for extended medical stabilization effort and resources are not considered in the current IMM simulations. In those missions where evacuation is not possible, the results may underpredict total medical events. This is because in the real world any event that meets evacuation criteria will either (1) resolve completely (and enable the crewmember to experience additional medical conditions), (2) result in chronic functional impairment of some kind (which also enables additional medical conditions), or (3) lead to death of a crewmember. It is beyond the scope of the model and underlying data to predict those pathways. Because every instance of potential evacuation results in one of these pathways, the loss of evacuation is likely to result in worsening risk posture for CHI and LOCL than results here predict.

Another operational parameter to consider is the effect of Extravehicular Activity (EVA) on medical risk. These estimates primarily look at *time* as a variable and are informed by the evidence base mostly gathered from ISS and Space Shuttle programs in addition to terrestrial medical evidence. In the case of the 14-day and 180-day DRMs, there are also two crewmembers who are modeled to perform three EVAs. This impacts only three medical conditions in the current study, but the real-world risk implications are more complicated. In this set of analyses, there is no other differentiation made for planetary surface EVAs. In an actual 730 day Mars DRM mission duration, there would likely be EVAs scheduled over ~30 days on the surface of the planet. For the sustaining Mars DRM over 1195 days, EVAs would be occurring on a cadence throughout several hundred days on the surface of the planet.

EVAs bring an increased likelihood of environmental and traumatic injury not captured with this simulation analysis. For example, musculoskeletal injuries occur in spaceflight due to several sources. A 2009 report suggested that the most common cause of musculoskeletal injuries was due to exercise (incidence 0.003 injuries per day aboard the ISS), but when crewmembers were performing EVA the incidence was 0.26 injuries per EVA mostly in hands and upper extremities^21,22^. This difference in musculoskeletal injury with EVA is not modeled by IMM. Because the current results represent a small number of EVAs compared to what is likely to be experienced in exploration missions, and the literature demonstrates a strong dependence of injury incidence on the number of EVAs, it is reasonable to assume that this contributes to under-prediction of total medical events as well as CHI, EVAC, and LOCL. This applies specifically when attempting to extrapolate results to Lunar or Mars mission planning where high numbers of EVAs are expected to be critical to mission success. Exploration atmospheres will be used to decrease pre-breathe time requirements for EVAs in lunar and planetary missions while maintaining the risk of decompression sickness at acceptable levels^23^. However, these atmospheres expose crew to chronic mild hypoxia and carry increased risk of acute mountain sickness, sleep disturbances, decreased exercise tolerance, and further impairment of the immune system, the extent of which is currently unknown^23,24^. As additional insight into mission attributes and plans become available, the IMM approach to EVAs may be modified to enable some estimation of this effect in future assessments. Some approaches have begun to provide a path forward^25^.

The final claim in the introduction was that deconditioning effects on the human body become more pronounced as mission duration increases. Real-world medical risk is dependent on some of these deconditioning effects. The assumption of constant incidence of medical conditions in long-duration missions is challenged by our knowledge of how deconditioning affects the body as mission duration increases. Examples of unimplemented capabilities include immune effects, food system effects, the effects of isolation and confinement, and other poorly defined effects from a hostile closed environment.

IMM assumes a constant incidence of medical conditions; however, for longer missions constant incidence may not be a realistic assumption given the changes in the immune system that occur over long-duration spaceflight. Immune system changes observed in spaceflight include alterations in T-cell and NK-cell function, elevated cytokines, and persistent inflammation. These changes appear to increase with mission duration and have resulted in increased hypersensitivity reactions and infections including viral reactivations^26^. Evidence suggests that immune changes can be effectively mitigated by ISS countermeasures including personal communication, exercise equipment and protocols, food quality and variety, nutritional supplementation, and schedule management^26^. As a result, the extent to which IMM predictions about medical condition incidences and risk are accurate will depend in part on the ability of an effective CHP system to maintain immune function, and not solely specific medical capabilities.

Immune health depends in part on adequate nutrition. We expect that food systems will be less effective for missions beyond LEO than they have been for ISS. This is because of the removal of resupply and the challenges associated with food preservation. Crews will lose access to fresh fruits and vegetables and studies have demonstrated that when food variety and acceptability are lacking, crews lose weight due to reduced calorie intake^8^. Mass, power, and volume restrictions are expected to limit refrigeration capabilities that help preserve food in missions^8^. These limitations are expected to have a negative impact on crew health and immune function.

Finally, psychological and behavioral changes experienced by exploration analog crews suggest that the incidence of depression, anxiety, and insomnia increase with mission duration^27–31^. These conditions are modeled in IMM but the assumption of constant incidence may underpredict when attempting to extrapolate to the 730 and 1195 day DRM cases. In addition, research into the secondary effects of the IMM medical conditions and their likely treatments suggests that both the symptoms and side effects have impacts on the behavioral and cognitive performance of the crew that may increase with mission duration^32^. These secondary effects are not considered by the CHI estimate.

All of these examples suggest that unimplemented capabilities in IMM can hinder the extrapolation of results shown here to missions beyond LEO. Some of these may be neutral in their effect on medical risk in the real world, but most will worsen medical risk posture. It is clear that none of them will serve to improve medical risk posture. It is possible, even likely, that technology improvements will improve risk reduction effects in the areas of medical capability, nutrition, and behavioral health support within the mass/power/volume/data bandwidth limitations inherent to a Mars mission prior to the first mission. However, speculating about the magnitude of possible future effects is beyond the scope of the model and this paper. From the best evidence-based information we have today, the net effect of these unimplemented capabilities and model limitations suggest that the LEO-specific estimates shown here are likely to underpredict the medical risk in real lunar or Mars missions.

### Conclusions

The results presented here more likely underpredict than overpredict medical risk contributions to total mission risk for missions beyond LEO. As the mission duration and distance from Earth increases, the uncertainty of overall risk grows to the point that it is not adequately represented by the uncertainty intervals shown here. From a mission planning perspective, over-prediction of medical risk can lead to too many mission resources dedicated to medical systems and less mass and volume for other mission needs. Under-prediction of medical risk can lead to insufficient resources dedicated to medical systems and worsened consequences from medical risk.

Even when medical risk is under-predicted, IMM estimates provide valuable information for mission planners. For instance, during the Space Shuttle Program, PRA estimates of the medical contribution to total mission risk were negligible because of the short duration of the missions and the protective effects of strong selection criteria^18^. As mission duration increases, IMM’s PRA estimates suggest that an initial Mars mission will carry a level of medical risk that is equal to or greater than the entire mission risk that astronauts faced on the Space Shuttle. Mars mission planners will need to incorporate a thoughtful and systematic approach to CHP System design to address the substantial medical risk. The estimated differences between the Unlimited ISS Medical Capability scenarios and the No Medical Capability scenarios highlight the impact that poor design or implementation could have on the medical risk carried by the astronauts, and thus the overall success of the mission.

The human system is like any other vehicle system. It requires maintenance and repair just as any other system in the vehicle. Historically, on shorter missions, NASA has relied on strong astronaut selection standards to mitigate medical risk. This analysis demonstrates that the ability of astronaut selection to mitigate medical risk diminishes as missions become longer, and a robust CHP System and effective Human System Integration becomes a necessity as longer-duration missions are planned. The requirements that inform a CHP system must be provided early in the systems engineering lifecycle for any vehicles designed to go to Mars or for long-duration stays at the Moon. Testing and validation options for a fielded CHP System are few and PRA modeling will be required to support CHP system design until more direct or observational testing can be performed during long-duration missions or analogs. The Artemis missions provide the best opportunity to test and validate a new CHP system in preparation for Mars exploration. IMM modeling provides strong evidence that medical risk increases with mission duration and distance from Earth. As a result, mission planners need to consider medical risk a significant factor in overall mission risk, and incorporate medical capabilities into mission planning early in the planning lifecycle.

## Supplementary information

Reporting Summary

Supplementary information

## Data Availability

The NASA Life Sciences Data Archive (LSDA) is the repository for all human and animal research data, including that associated with this study. LSDA has a public-facing portal where data requests can be initiated (https://lsda.jsc.nasa.gov/Request/dataRequestFAQ). The LSDA team provides the appropriate processes, tools, and secure infrastructure for archival of experimental data and dissemination while complying with applicable rules, regulations, policies, and procedures governing the management and archival of sensitive data and information. The LSDA team enables data and information dissemination to the public or to authorized personnel either by providing public access to information or via an approved request process for information and data from the LSDA in accordance with NASA Human Research Program and JSC Institutional Review Board direction.

## Code availability

The NASA Life Sciences Data Archive (LSDA) is the repository for all human and animal research data, including that associated with this study. LSDA has a public-facing portal where code requests for IMM can be initiated (https://lsda.jsc.nasa.gov/Request/dataRequestFAQ). IMM version 4.1 was used for this study. The IMM code is owned by NASA Johnson Space Center with continuing funding through the Crew Health and Performance Program and operated by KBR.
