# Supplementary material for: Estimating medical risk in human spaceflight
Source: NPJ Microgravity. 2022 Mar 31;8:8. doi: 10.1038/s41526-022-00193-9 (PMC8971481; doi:10.1038/s41526-022-00193-9)
Supplement: Supplementary file 2 — Supplementary information [file 41526_2022_193_MOESM2_ESM.pdf]

Table 2: Medical Conditions in iMED by category (iMED_20170221).

ENVIRONMENTAL

Acute Radiation Syndrome
Altitude Sickness
Barotrauma (ear/sinus block)
Burns secondary to Fire
Decompression Sickness Secondary to EVA
Eye Chemical Burn
Headache (CO_2 induced)
Smoke Inhalation
Toxic Exposure: Ammonia

INJURY/TRAUMA

Abdominal Injury
Acute Compartment Syndrome
Ankle Sprain/Strain
Back Sprain/Strain
Chest Injury
Dental: Avulsion (tooth loss)
Elbow Dislocation
Elbow Sprain/Strain
Eye Irritation/Abrasion
Eye Penetration (foreign body)
Finger Dislocation
Fingernail Delamination Secondary to EVA
Head Injury
Hip Sprain/Strain
Hip/Proximal Femur Fracture
Knee Sprain/Strain
Lower Extremity Stress Fracture
Lumbar Spine Fracture
Neck Sprain/Strain
Neurogenic Shock
Paresthesias Secondary to EVA
Shoulder Dislocation
Shoulder Sprain/Strain
Skin Abrasion
Skin Laceration
Traumatic Hypovolemic Shock
Wrist Fracture
Wrist Sprain/Strain

MEDICAL ILLNESS

Abdominal Wall Hernia
Abnormal Uterine Bleeding
Acute Angle-Closure Glaucoma
Acute Arthritis
Acute Cholecystitis/Biliary Colic
Acute Diverticulitis
Acute Pancreatitis
Acute Prostatitis
Acute Sinusitis
Allergic Reaction (mild to moderate)
Anaphylaxis
Angina/Myocardial Infarction

MEDICAL ILLNESS (continued)

Anxiety
Appendicitis
Atrial Fibrillation/Atrial Flutter
Back Pain (space adaptation)
Behavioral Emergency
Cardiogenic Shock Secondary to Myocardial Infarction
Choking/Obstructed Airway
Constipation (space adaptation)
Dental: Exposed Pulp
Dental Caries
Dental: Abscess
Dental: Crown Loss
Dental: Filling Loss
Depression
Diarrhea
Eye Corneal Ulcer
Eye Infection
Gastroenteritis
Headache (Late)
Headache (space adaptation)
Hearing Loss
Hemorrhoids
Herpes Zoster Reactivation (shingles)
Hypertension
Indigestion
Influenza
Insomnia (space adaptation)
Medication Overdose/Adverse Reaction
Mouth Ulcer
Nasal Congestion (space adaptation)
Nephrolithiasis
Nose bleed (space adaptation)
Otitis Externa
Otitis Media
Pharyngitis
Respiratory Infection
Retinal Detachment
Seizures
Sepsis
Skin Infection
Skin Rash
Sleep Disorder
Small Bowel Obstruction
Space Motion Sickness (space adaptation)
Stroke (Cerebrovascular Accident)
Sudden Cardiac Arrest
Urinary Incontinence (space adaption)
Urinary Retention (space adaptation)
Urinary Tract Infection
Vaginal Yeast Infection
Space Adaptation Neuro-Ocular Syndrome (SANS) (space adaptation)

The Design Reference Missions (DRMs) referenced here are in Table 1 in the main paper.

Table 3: Crew profile for DRM 1 (14 Days)

	Sex	CAC	Crowns	Contacts	Prior Abdominal Surgery	EVA
Crew 1	Male	No	Yes	No	No	No
Crew 2	Male	Yes	No	No	Yes	Yes (3)
Crew 3	Female	No	No	Yes	No	Yes (3)
Crew 4	Female	No	No	No	Yes	No
Crew 5	Male	No	No	Yes	No	No
Crew 6	Male	No	Yes	No	No	No
Crew 7	Female	No	No	Yes	No	No

Table 4: Crew profile for DRMs 2 (21 days), 3 (42 days), 5 (365 days), 6 (730 days), and 7 (1195 days)

	Sex	CAC	Crowns	Contacts	Prior Abdominal Surgery	EVA
Crew 1	Male	No	Yes	No	No	No
Crew 2	Male	Yes	No	No	No	No
Crew 3	Female	No	No	Yes	No	No
Crew 4	Female	No	No	No	Yes	No

Table 5: Crew profile for DRM 4 (180 days)

	Sex	CAC	Crowns	Contacts	Prior Abdominal Surgery	EVA
Crew 1	Male	No	No	Yes	No	No
Crew 2	Male	Yes	No	No	No	Yes (3)
Crew 3	Female	No	No	Yes	No	Yes (3)
Crew 4	Female	No	Yes	No	Yes	No
Crew 5	Male	No	No	Yes	No	No
Crew 6	Male	No	Yes	No	No	No

Table 6: Total Medical Events (TME) – tabular data for Figure 1.

	Medical Capability	TME	95% CI	
**14 day 7 crew DRM**	Limited ISS	28.97	21	38
	Unlimited ISS	28.97	21	38
	No Medical	28.66	21	37
**21 day 4 crew DRM**	Limited ISS	18.18	12	25
	Unlimited ISS	18.18	12	25
	No Medical	17.95	11	25
**42 day 4 crew DRM**	Limited ISS	25.47	17	34
	Unlimited ISS	25.47	17	34
	No Medical	24.94	17	34
**180 day 6 crew DRM**	Limited ISS	104.18	85	124
	Unlimited ISS	104.21	85	124
	No Medical	97.14	73	120
**365 day 4 crew DRM**	Limited ISS	126.79	103	150
	Unlimited ISS	126.98	103	150
	No Medical	109.78	69	143
**730 day 4 crew DRM**	Limited ISS	237.43	190	273
	Unlimited ISS	239.62	195	274
	No Medical	180.36	94	253
**1195 day 4 crew DRM**	Limited ISS	369.95	285	424
	Unlimited ISS	381.22	301	429
	No Medical	244.04	106	376

Table 7: Crew Health Index (CHI) – tabular data for Figure 2.

	Medical Capability	CHI	95% CI	
**14 day 7 crew DRM**	Limited ISS	96.04	92.51	98.16
	Unlimited ISS	96.03	92.40	98.16
	No Medical	90.58	78.37	95.81
**21 day 4 crew DRM**	Limited ISS	96.99	92.99	98.84
	Unlimited ISS	96.99	92.95	98.84
	No Medical	90.23	69.75	96.89
**42 day 4 crew DRM**	Limited ISS	97.36	92.90	98.99
	Unlimited ISS	97.36	92.87	98.99
	No Medical	86.13	65.24	94.80
**180 day 6 crew DRM**	Limited ISS	95.01	84.43	98.49
	Unlimited ISS	95.05	84.59	98.50
	No Medical	60.72	45.02	72.46
**365 day 4 crew DRM**	Limited ISS	93.02	76.17	98.32
	Unlimited ISS	93.36	76.69	98.43
	No Medical	41.44	25.63	54.39
**730 day 4 crew DRM**	Limited ISS	86.00	68.28	94.89
	Unlimited ISS	90.39	72.11	97.38
	No Medical	23.78	14.00	32.58
**1195 day 4 crew DRM**	Limited ISS	72.35	55.04	84.16
	Unlimited ISS	86.87	67.72	95.75
	No Medical	14.82	8.64	20.52

Table 8: Evacuation (EVAC) – tabular data for Figure 3.

	Medical Capability	EVAC	95% CI	
**14 day 7 crew DRM**	Limited ISS	0.0032	0.0029	0.0036
	Unlimited ISS	0.0031	0.0028	0.0035
	No Medical	0.2044	0.2017	0.2069
**21 day 4 crew DRM**	Limited ISS	0.0027	0.0024	0.0030
	Unlimited ISS	0.0026	0.0023	0.0029
	No Medical	0.1400	0.1379	0.1421
**42 day 4 crew DRM**	Limited ISS	0.0109	0.0102	0.0115
	Unlimited ISS	0.0106	0.0100	0.0113
	No Medical	0.2035	0.2009	0.2059
**180 day 6 crew DRM**	Limited ISS	0.0547	0.0532	0.0560
	Unlimited ISS	0.0468	0.0454	0.0481
	No Medical	0.6469	0.6439	0.6498
**365 day 4 crew DRM**	Limited ISS	0.0755	0.0737	0.0771
	Unlimited ISS	0.0521	0.0507	0.0535
	No Medical	0.7393	0.7365	0.7420
**730 day 4 crew DRM**	Limited ISS	0.2121	0.2096	0.2146
	Unlimited ISS	0.0951	0.0934	0.0969
	No Medical	0.9243	0.9227	0.9259
**1195 day 4 crew DRM**	Limited ISS	0.4330	0.4300	0.4358
	Unlimited ISS	0.1436	0.1415	0.1458
	No Medical	0.9840	0.9832	0.9848

Table 9: Loss of Crew Life (LOCL) – tabular data for Figure 4.

	Medical Capability	LOCL	95% CI	
**14 day 7 crew DRM**	Limited ISS	0.0005	0.0003	0.0006
	Unlimited ISS	0.0005	0.0004	0.0006
	No Medical	0.0022	0.0019	0.0025
**21 day 4 crew DRM**	Limited ISS	0.0003	0.0002	0.0005
	Unlimited ISS	0.0003	0.0002	0.0005
	No Medical	0.0015	0.0013	0.0018
**42 day 4 crew DRM**	Limited ISS	0.0007	0.0006	0.0009
	Unlimited ISS	0.0007	0.0006	0.0009
	No Medical	0.0033	0.0030	0.0037
**180 day 6 crew DRM**	Limited ISS	0.0049	0.0045	0.0054
	Unlimited ISS	0.0048	0.0044	0.0053
	No Medical	0.0194	0.0185	0.0202
**365 day 4 crew DRM**	Limited ISS	0.0059	0.0054	0.0063
	Unlimited ISS	0.0059	0.0054	0.0063
	No Medical	0.0239	0.0229	0.0248
**730 day 4 crew DRM**	Limited ISS	0.0126	0.0119	0.0133
	Unlimited ISS	0.0124	0.0117	0.0131
	No Medical	0.0425	0.0413	0.0439
**1195 day 4 crew DRM**	Limited ISS	0.0214	0.0206	0.0224
	Unlimited ISS	0.0209	0.0200	0.0217
	No Medical	0.0571	0.0558	0.0585
